# Supplementary material for: Identification and functional analysis of non-coding regulatory small RNA FenSr3 in Bacillus amyloliquefaciens LPB-18
Source: PeerJ. 2023 May 15;11:e15236. doi: 10.7717/peerj.15236 (PMC10194069; doi:10.7717/peerj.15236)
Supplement: Supplemental Information 4 [file peerj-11-15236-s004.zip › KO/CK-vs-T1_map/map04940.html]

KEGG PATHWAY: Type I diabetes mellitus - Reference pathway


|  |  |
| --- | --- |
| **Type I diabetes mellitus - Reference pathway** |  |

[
Pathway menu
| Organism menu
| Pathway entry
| Show description
| User data mapping
]

Type I diabetes mellitus is a disease that results from autoimmune destruction of the insulin-producing beta-cells. Certain beta-cell proteins act as autoantigens after being processed by antigen-presenting cell (APC), such as macrophages and dendritic cells, and presented in a complex with MHC-II molecules on the surface of the APC. Then immunogenic signals from APC activate CD4+ T cells, predominantly of the Th1 subset. Antigen-activated Th1 cells produce IL-2 and IFNgamma. They activate macrophages and cytotoxic CD8+ T cells, and these effector cells may kill islet beta-cells by one or both of two types of mechanisms: (1) direct interactions of antigen-specific cytotoxic T cells with a beta-cell autoantigen-MHC-I complex on the beta-cell, and (2) non-specific inflammatory mediators, such as free radicals/oxidants and cytokines (IL-1, TNFalpha, TNFbeta, IFNgamma).
Type I diabetes is a polygenic disease. One of the principle determining genetic factors in diabetes incidence is the inheritance of mutant MHC-II alleles. Another plausible candidate gene is the insulin gene.

|  |  |  |
| --- | --- | --- |
| Reference pathway | 184% 150% 122% 100% 82% 67% 55% | 图片下载 |
